# Linking Individual Learning Styles to Approach-Avoidance Motivational Traits and Computational Aspects of Reinforcement Learning

**DOI:** 10.1371/journal.pone.0166675

**Published:** 2016-11-16

**Authors:** Kristoffer Carl Aberg, Kimberly C. Doell, Sophie Schwartz

**Affiliations:** 1 Department of Neuroscience, Faculty of Medicine, University of Geneva, Geneva, Switzerland; 2 Swiss Center for Affective Sciences, University of Geneva, Geneva, Switzerland; 3 Geneva Neuroscience Center, University of Geneva, Geneva, Switzerland; Brain and Spine Institute (ICM), FRANCE

## Abstract

Learning how to gain rewards (approach learning) and avoid punishments (avoidance learning) is fundamental for everyday life. While individual differences in approach and avoidance learning styles have been related to genetics and aging, the contribution of personality factors, such as traits, remains undetermined. Moreover, little is known about the computational mechanisms mediating differences in learning styles. Here, we used a probabilistic selection task with positive and negative feedbacks, in combination with computational modelling, to show that individuals displaying better approach (vs. avoidance) learning scored higher on measures of approach (vs. avoidance) trait motivation, but, paradoxically, also displayed reduced learning speed following positive (vs. negative) outcomes. These data suggest that learning different types of information depend on associated reward values and internal motivational drives, possibly determined by personality traits.

## Introduction

Much of human behaviour is directed towards maximizing rewards (via approach behaviour) and minimizing punishments (via avoidance behaviour). While individuals display differences in the ability to learn from rewards (approach learning) and punishments (avoidance learning), the link between approach and avoidance learning and the general expression of approach and avoidance behaviours is not well established.

A frequently used paradigm in the literature on approach and avoidance learning is the probabilistic selection task (PST; [1]), in which participants first learn reward probabilities (i.e. the frequency of positive and negative outcomes) associated with different symbols, and then use the learned reward probabilities to guide decision making in a subsequent testing phase (i.e. the discrimination between novel pairs of symbols; [1]). Some individuals, ‘approach learners’, are better at selecting symbols previously associated with frequent positive outcomes, while others, ‘avoidance learners’, express the reverse trend, i.e. enhanced rejection of symbols previously associated with frequent negative outcomes. The expression of different approach and avoidance learning styles has been related to factors such as particular gene polymorphisms [1,2], different levels of dopamine function [1,3–5], hemispheric asymmetries in dopamine function [6–8], age [9], and individual striatal D1 and D2 receptor function [10,11]. The impact of these factors on approach and avoidance learning have been explained using both classical reinforcement learning models [12] and more advanced neural network models [13,14].

Yet, the link between approach and avoidance learning styles and the general expression of approach and avoidance behaviours, as indexed by personality traits, still remains unclear. For example, avoidance learning has been shown to correlate positively with harm avoidance [4], but also positively with novelty seeking, a trait commonly associated with approach tendencies [15]. Adding to these discrepant data, in a recent study [16], no correlations were reported between approach and avoidance learning and personality traits, as estimated using the Behavioural Inhibition System/Behavioural Activation System scales (BIS/BAS scales; [17]). Clarifying the relationship between personality traits and the learning of different types of information may not only improve our understanding of the aetiology of disorders characterized by the extreme expression of approach and avoidance behaviours (i.e. anxiety, depression, and addiction disorders, see [18–21]), but could also help improve educational programs by highlighting the need for tailoring learning contexts based on each person’s sensitivity to rewarding and punishing incentives.

The present study was designed to investigate the relationship between approach and avoidance learning styles and personality traits pertaining to approach and avoidance behaviours, as well as the computational mechanisms mediating the expression of different learning styles. In brief, 34 participants performed the PST to assess approach and avoidance learning, and the expression of individual approach and avoidance motivational traits were estimated using the Behavioural Inhibition System/Behavioural Activation System scales (BIS/BAS scales; [17,22]) and the Sensitivity to Punishment and Sensitivity to Reward Questionnaire (SPSRQ; [23,24]). Additionally, a classical reinforcement learning model was implemented to investigate the computational mechanisms mediating individual differences in learning styles [12]. Computational approaches are particularly useful when studying individual differences in learning because they allow for the reduction of complex learning behaviours into a few interpretable parameters, such as the rate of learning different types of information, which can then be compared between individuals displaying, for example, different learning styles or personality traits.

The results show that approach learners, i.e. participants displaying better learning from positive (vs. negative) outcomes, display increased trait approach as well as reduced trait avoidance, as compared to avoidance learners. These results evidence a clear link between an individual’s approach and avoidance learning style and the tendency to display approach and avoidance behaviours. Moreover, the computational approach revealed that approach learners learned relatively slower and faster following positive and negative outcomes, respectively, while avoidance learners displayed the reverse trend. This apparently paradoxical finding could highlight a mechanism which allows slow integration and learning of information that is congruent with an individual’s trait, eventually leading to more stable and persistent memories which could contribute to the maintenance and reinforcement of behavioural predispositions.

## Material and Methods

### Ethics statement

All participants provided written informed consent prior to participating in this study. This study was carried out in accordance with the latest version of the Declaration of Helsinki and was approved by the Ethical Committee of the Geneva University Hospitals.

### Participants

Forty-two healthy participants with no previous history of neurological or psychological disorders participated in the study. Data from eight participants had to be excluded for the following reasons: failure to follow task instructions (n = 4) and failure to reach the performance criteria in the probabilistic selection task (n = 4, see below). Finally, data from 34 right handed and native French speaking participants [14 females; average age 23.41 ± 0.78 years ± SEM] were included in the analyses.

### Probabilistic selection task (PST)

**Fig 1:**
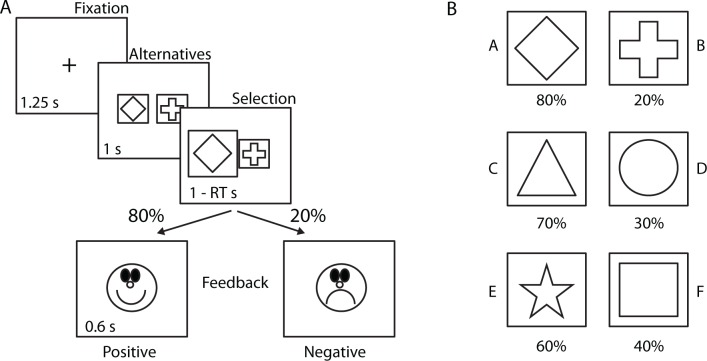
Probabilistic Selection Task (PST). A. One training trial in the PST. After fixation, two symbols were presented and participants selected one symbol within 1s. After 1s positive or negative feedback was presented based on the reward probability associated with the selected symbol. RT = response time. B. Reward probabilities associated with each pair and symbol. The symbols associated with each reward probability were randomized between participants.

All participants performed a probabilistic selection task (PST) used to assess approach-avoidance learning [1]. In the PST, participants learned symbol-values in a training phase by associating each symbol with different reward probabilities. In each trial, one of three pairs of symbols (AB, CD, or EF) was presented and participants selected one symbol by pressing its corresponding button with the right hand (Fig 1A).

After selection, a positive or negative smiley face was presented. The type of feedback presented depended on the reward probability associated with each symbol (Fig 1B). For example, selecting the A symbol in an AB pair resulted in positive feedback 80% of the time while selecting the B symbol would result in negative feedback 80% of the time (reward probabilities for symbols in CD and EF pairs were 70/30% and 60/40%, respectively). During training participants were instructed to increase the number of outcomes with happy smiley faces while decreasing the number of outcomes with sad smiley faces. To ensure that learning had occurred, participants were required to reach predefined criteria (selecting A and C symbols 60 and 55% of the time, respectively, within one block of 60 trials) before continuing to the next phase of the task (for a similar procedure see [3,6]). Data from participants failing to reach the criteria within 45 minutes of training were excluded from further analyses (n = 4). Next, participants underwent a test phase in which they were presented with twelve additional novel pairs (AC, AD, AE, AF, BC, BD, BE, BF, CE, CF, DE, and DF), created by mixing the symbols from the original trained pairs (AB, CD, and EF). This test phase was similar to the training phase with the exception that no feedback was presented to prevent further learning of the new pairs. Participants were instructed to perform the task as well as possible and to trust their instinct, or guess, when uncertain. Approach and avoidance learning were defined during the test phase with the novel pairs as the proportion of trials in which the A symbol (most frequently rewarded during training) was selected and the B symbol (most frequently punished) was rejected, respectively [1].

### Computational approach

A computational approach was adopted to test the impact of different learning styles on computational reinforcement learning mechanisms. Frank and Claus [14] suggested that two learning systems may account for differences in learning behaviour. The first system relates to rapid updating of reward information in working memory (WM), while the second system is related to the slow integration of reward information and habitual responding. It was recently shown that computational parameters indexing these two learning systems were influenced by different gene-polymorphisms related to striatal and prefrontal dopamine function [12]. To test whether individual approach-avoidance learning styles in the present study could be determined by one or both of the abovementioned learning systems, we implemented the modelling approach suggested by Frank, Moustafa, Haughey, Curran, and Hutchison [12]:

Each symbol *i* is assigned a value *Q*_*i*_ which depends on its feedback history. Specifically, the value *Q*_*i*_ is updated each time the corresponding symbol has been selected: *Q*_*i*_(*t* + 1) = *Q*_*i*_(*t*) + *α*_*Approach*_[*r*(*t*) − *Q*_*i*_(*t*)]_+_ + *α*_*Avoid*_[*r*(*t*) − *Q*_*i*_(*t*)]_−_ where *Q*_*i*_(*t*) is the value for the selected symbol *i* in trial *t*, *α*_*Approach*_ and *α*_*Avoid*_ are the learning rates for positive and negative outcomes (denoted by the +, and–subscripts, respectively), and *r*(*t*) is the reward outcome (set to 1 for positive outcomes and 0 for negative outcomes). The probability of selecting a specific symbol is estimated through a softmax choice probability rule: $p_{A}(t)=\frac{e^{\frac{Q_{A}(t)}{\beta }}}{e^{\frac{Q_{A}(t)}{\beta }}+e^{\frac{Q_{B}(t)}{\beta }}}$. In this example, *p*_*A*_ is the probability of selecting symbol A in an AB pair. The *β* controls ‘exploit vs. explore’ behavior during the training. When this parameter is small, the symbol with the highest Q value is most likely selected (exploitation) while a large value leads to selections less dependent on the symbol’s value (exploration). The three parameters *α*_*Approach*_, *α*_*Avoid*_, and *β* were fit to each participant’s behaviour by minimizing the negative log likelihood estimate (LLE): $LLE=-log(\prod_{t}^{n}p_{i}(t))$, where *p*_*i*_(*t*) is the probability of selecting symbol *i* in trial *t*. The function of the WM system and the habitual learning system can then be assessed by fitting the parameters to performance during the training and testing phase, respectively [12]. Fitting the model to behaviour during the test phase is accomplished by assuming that decision making during the test phase is determined by the Q-values obtained at the end of training. This is a plausible assumption because removal of feedback during the testing phase prevents further learning of reward contingencies.

In addition to the approach/avoidance model just described a canonical model with only one learning rate was fit to behavioural data. Their respective fits were compared using Akaike’s Information Criterion (AIC; [25]) which accounts for different numbers of fitted parameters (*k*): *AIC* = 2 * *LLE* + 2 * *k*. Additionally, a parameter-free “null-model”, assuming that all choices are random and equiprobable, was used to compute a standardized metric of model fit. This pseudo-*R*^2^ statistic was defined as the improvement from a null model to the fitted model, i.e. pseudo-R^2^ = 1—*LLE*_*fitted*_/*LLE*_*random*_, where *LLE*_*random*_ is the log-likelihood estimate under the random choice model and *LLE*_*fitted*_ is the log-likelihood estimate under the fit model [26,27].

### Questionnaires

To determine whether participants displaying different learning styles also expressed differences in motivational traits pertaining to approach and avoidance behaviours, all participants filled out French versions of the Sensitivity to Punishment (SP) and Sensitivity to Reward (SR) Questionnaire (SPSRQ; [23,24]) and the Behavioural Inhibition System (BIS) and Behavioural Activation System (BAS) scales (BIS/BAS scales; [17,22]). Z-scores were used to account for different number of items in the BIS, BAS, SP, and SR subscales. A total BAS z-score was calculated as the z-score for the sum of the Drive, Fun Seeking, and Reward Responsiveness subscales [8].

### Statistics

Statistical analyses were conducted using Analyses of Variance (ANOVAs) and *t*-tests. The Anderson-Darling test was used to ensure that data did not deviate significantly from the normal distribution [28]. Correlations were calculated using the Spearman’s ρ.

## Results

### Behaviour

As in previous studies [15,29], participants were divided into two groups based on whether they were better at selecting A than rejecting B during the test phase (approach learners; n = 21) while avoidance learners (n = 13) were those displaying the opposite trend.

#### Training phase

**Fig 2:**
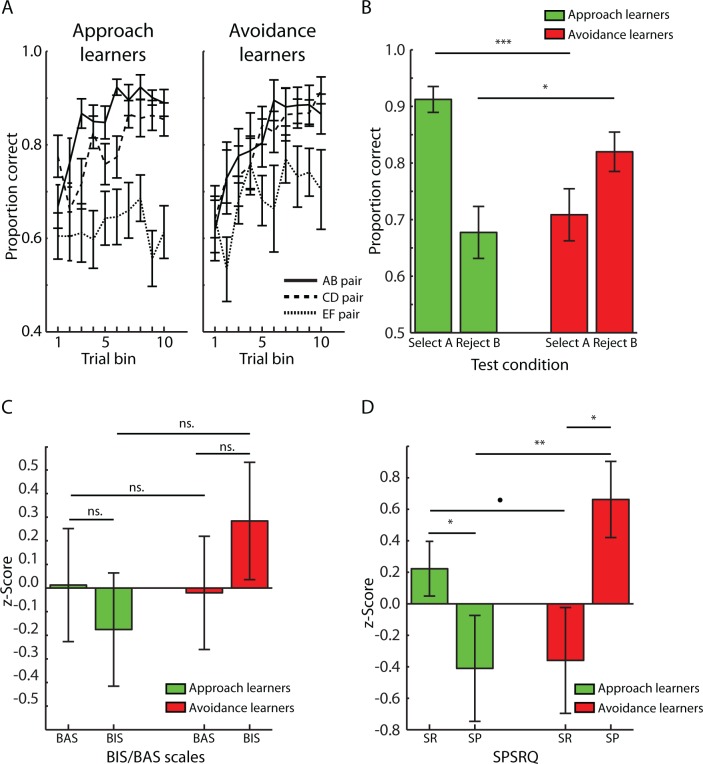
Behavioural data (Mean ± SEM). A. Performance as a function of training. Performance improved equally for participants in the different groups as training progressed. B. Approach and avoidance performance during testing. Approach learners were relatively better at selecting the A symbol, as compared to rejecting the B symbol, while avoidance learners displayed the reverse trend. Importantly, this interaction simply reflects the assignment of participants to different groups based on their relative performance on selecting the A-rejecting the B symbol. However, approach learners were also better at selecting the A symbol as compared to avoidance learners, while the B symbol was more frequently rejected by avoidance learners. C. Approach and avoidance learners did not differ in BAS nor BIS scores. D. Approach and avoidance learners scored higher on reward sensitivity (SR) and punishment sensitivity (SP), respectively. BAS = behavioural activation system, BIS = behavioural inhibition system, SP = sensitivity to punishment, SR = sensitivity to reward, SPSRQ = Sensitivity to Punishment and Sensitivity to Reward Questionnaire. • p< 0.10, * p < 0.05, ** p < 0.01,*** p < 0.001, ns. = not significant (p>0.05).

The two groups of learners did not differ in the number of training blocks needed to reach the criteria [mean number of blocks: approach learners = 4.191 ± 3.669 (SEM); avoidance learners = 3.000 ± 2.345 (SEM); *t*(32) = 1.042, p = 0.305]. Group difference in training performance was further investigated by logistic regression analysis in which trial numbers were used as performance predictors for each pair (AB, CD, EF). The resulting coefficients were entered into a mixed-effect ANOVA with Group (approach, avoidance learners) as a between-subjects factor and Pair (AB, CD, EF) as a within-subjects factor. There were no significant effects of Group [F(1,32) = 1.156, p = 0.290] or Pair [F(2,64) = 0.419, p = 0.660], nor Group x Pair interaction [F(2,64) = 0.038, p = 0.968]. Moreover, post-hoc paired *t*-tests, corrected for three multiple comparisons, showed that regression coefficients were significantly larger than 0 for AB and CD pairs [mean coefficient: AB-pairs = 0.017 ± 0.004 (SEM), *t*(33) = 4.408, p < 0.001; CD-pairs = 0.015 ± 0.005 (SEM), *t*(33) = 2.946, p = 0.018], but not for EF-pairs [mean coefficient: EF-pairs = 0.011 ± 0.006 (SEM), *t*(33) = 2.013, p = 0.156]. Together, these results indicate that learning occurred, but did not differ between the two groups of learners. Performance as a function of training is displayed in Fig 2A. Of note, for display purposes training performance was averaged across trials in ten equally sized bins because individuals differed in the number of trials needed to reach the criteria.

#### Testing phase

Selection rates of A-symbols and rejection rates of B-symbols during the testing phase are displayed in Fig 2B and reported in Table 1. A mixed-effects ANOVA with between-subjects factor Group (approach, avoidance learner) and within-subject factor Symbol (A, B) revealed a significant main effect of Symbol [F(1,32) = 12.917, p = 0.001] because participants were generally better at selecting the A symbol as compared to avoiding the B symbol. There was also a significant Group x Symbol interaction [F(1,32) = 34.750, p < 0.001] because approach learners were better at selecting the A symbol as compared to rejecting the B symbol [*t*(20) = 6.022, p < 0.001], while avoidance learners were better at rejecting the B symbol as compared to selecting the A symbol [*t*(12) = 2.797, p = 0.050]. Of note, these are purely descriptive results which are the product of assigning participants to different groups based on the relative difference between selecting A vs. rejecting B symbols. However, approach learners selected the A symbol more frequently than avoidance learners [*t*(32) = 4.409, p < 0.001] while avoidance learners were better at rejecting the B symbol [*t*(32) = 2.433, p = 0.020]. These two latter results are not simply due to how participants were assigned to different groups, because, for example, participants could be assigned to the same groups if all participants performed equally well on trials with A-symbols but differently on trials with B-symbols (or vice versa). Finally, overall performance did not differ between groups, as indicated by a non-significant effect of Group [F(1,32) = 0.488, p = 0.490]. These results are in accordance with, and extend, previous findings indicating that the balance between approach and avoidance learning may be determined by inter-individual factors such as gene expression [2,12] and striatal dopamine function [5,10]. In summary, these results indicate that participants can be characterized as belonging to one of two groups of learners, which differ in approach and avoidance learning but not overall ability to learn reward/punishment probabilities.

**Table 1:** Selection rates during testing and trait scores. Mean ± SEM.

	Learning type	
	Approach (n = 21)	Avoidance (n = 13)
**Selection rates**		
Select A	0.912±0.023	0.709±0.046
Reject B	0.677±0.041	0.820±0.035
**BIS/BAS scales**		
BAS	0.013±0.239	-0.020±0.240
BIS	-0.176±0.173	0.284±0.249
**SPSRQ**		
SR	0.223±0.239	-0.360±0.336
SP	-0.410±0.187	0.663±0.242

BAS is the z-scored sum of the BAS subscales (i.e. Drive, Fun Seeking, and Reward Responsiveness), while BIS is the z-scored BIS subscale, of the Behavioural Inhibition System and Behavioural Activation System scales (BIS/BAS scales; [17,22]). SR and SP refer to the z-scored values on the SR and SP subscales, respectively, of the Sensitivity to Punishment and Sensitivity to Reward Questionnaire (SPSRQ; [23,24]).

### Questionnaires

Scores on the BIS/BAS scales are displayed in Fig 2C and reported in Table 1. A mixed-effects ANOVA with between-subject factor Group (approach, avoidance learner) and within-subject factor BIS/BAS scale (BIS, BAS) revealed no significant main effects or interaction [Fig 2C; all p-values > 0.420]. By contrast, a similar ANOVA with within-subject factor SPSRQ (SP, SR) revealed a significant interaction with Group [Fig 2D; F(1,32) = 13.032, p = 0.001] because approach learners displayed relatively higher SR than SP [*t*(20) = 2.206, p = 0.039] and avoidance learners displayed the reverse trend, i.e. relatively higher SP than SR [*t*(12) = 2.284, p = 0.041]. Moreover, avoidance learners, as compared to approach learners, displayed significantly higher SP [*t*(32) = 3.528, p = 0.001] while approach learners, as compared to avoidance learners, displayed marginally higher SR [*t*(32) = 1.698, p = 0.098]. SPSRQ scores are displayed in Fig 2D and reported in Table 1. These results demonstrate a significant link between approach/avoidance learning styles and the relative expression of approach/avoidance motivational traits.

### Computational model

To determine the computational mechanisms contributing to different learning styles, we adopted a computational approach which posits that reinforcement learning is under the control of two learning systems. The WM system controls the rapid updating of reward information, while the habitual responding system relies on the slow integration of reward information [3,12]. To assess the function of the WM system and the habitual system, a reinforcement learning model was fit to each participant’s data during the training and the testing phase, respectively [12].

#### Working memory (WM) learning system

**Fig 3:**
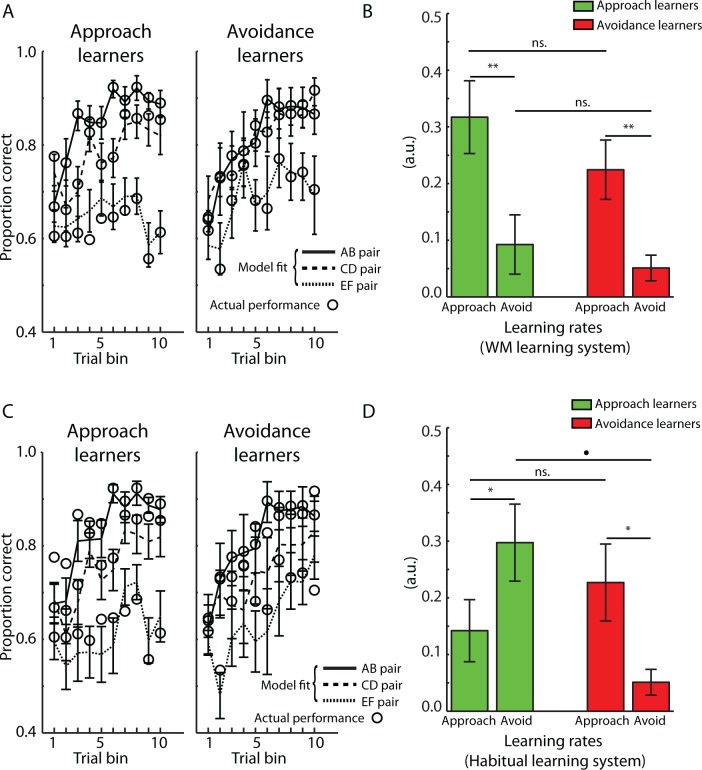
Model-fitted data (Mean ± SEM). A. To assess approach and avoidance learning in the working memory (WM) system, the approach/avoidance model was fit to participants’ behaviour during the training phase [12]. Model-derived proportion of correct selections during the training phase is displayed by the lines, while actual behaviour is displayed by the circles. B. Approach and avoidance learning rates for the WM system. Approach and avoidance learners did not differ in the learning rates for the WM system. C. To assess approach and avoidance learning in the Habitual learning system, the approach/avoidance model was fit to participants’ behaviour during the testing phase [12]. Model-derived proportion of correct selections during the training phase is displayed by the lines while actual behaviour is displayed by the circles. D. Approach and avoidance learning rates for the Habitual learning system. Approach learners displayed relatively slower learning rates from positive feedback (*α*_*Approach*_) as compared to negative feedback (*α*_*Avoid*_), while avoidance learners displayed the reverse trend. *α*_*Approach*_ = learning rate following positive feedback, *α*_*Avoid*_ = learning rate following negative feedback. • p< 0.10, * p < 0.05, ** p < 0.01, ns. = not significant (p>0.05).

The fitted parameters of the canonical model and the approach/avoidance model are reported in Table 2. A paired *t*-test on the average AIC scores revealed that the approach/avoidance model provided a significantly better fit to behaviour as compared to the canonical model [*t*(33) = 2.4571, p = 0.0194]. The fit of the approach/avoidance model is displayed in Fig 3A. The model-derived parameters (i.e. *α*_*Approach*_, *α*_*Avoid*_, and *β*) were compared between the different types of learners (see Table 3). A mixed-effects ANOVA with between-subjects factor Group (approach, avoidance learner) and within-subject factor Learning rate (*α*_*Approach*_, *α*_*Avoid*_) revealed a significant main effect of Learning rate [F(1, 32) = 23.968, p < 0.001], because *α*_*Approach*_ was significantly larger than *α*_*Avoid*_ (see Fig 3B). This result indicates that symbol values were updated more rapidly following positive outcomes, as compared to negative outcomes. By contrast, there was no main effect of Group or Group x Learning rate interaction [both p-values > 0.240]. Moreover, the exploration/exploitation parameter *β* did not differ between the groups [t(32) = 0.317, p = 0.754].

**Table 2:** Model fits. Mean ± SEM.

Model	-LLE	AIC	Pseudo-R^2^	*α*	*α*_*Approach*_	*α*_*Avoid*_	β
**Working memory**							
**Random choice**	240.76±22.860	240.767±45.720	-	-	-	-	-
**Canonical**	182.068±23.126	186.068±46.252	0.291±0.028	0.152±0.024	-	-	0.286±0.043
**Approach/avoidance**	174.586±22.641	180.586±45.282	0.320±0.029	-	0.282±0.045	0.077±0.019	0.258±0.027
**Habitual**							
**Random choice**	79.467±0.581	79.467±1.162	-	-	-	-	-
**Canonical**	55.801±2.398	59.801±4.796	0.297±0.030	0.223±0.056	-	-	0.229±0.037
**Approach/avoidance**	51.384±2.696	57.384±5.392	0.354±0.036	-	0.175±0.043	0.221±0.054	0.161±0.032

LLE is the log-likelihood estimate. AIC is the Akaike Information Criterion. α denotes learning rates and β the trade-off between exploration and exploitation. Working memory and Habitual refer to two different learning systems which can be assessed by fitting model parameters to behavioural data during the training and testing phase, respectively [12].

**Table 3:** Fitted model parameters separately for approach and avoidance learners. Mean ± SEM.

	Learning type	
Model	Approach(n = 21)	Avoid(n = 13)
**Working memory**		
***α***_***Approach***_	0.317±0.064	0.224±0.052
***α***_***Avoid***_	0.093±0.028	0.051±0.023
**β**	0.247±0.034	0.265±0.039
**Habitual**		
***α***_***Approach***_	0.142±0.055	0.227±0.068
***α***_***Avoid***_	0.297±0.078	0.098±0.050
**β**	0.224±0.062	0.122±0.033

“Working memory” and “Habitual” refers to the two different learning systems which can be assessed by fitting model parameters to behavioural data during the training and testing phase, respectively [12].

#### Habitual learning system

Fitted parameters for the two different models are shown in Table 2. As for the WM system, the approach/avoidance model provided the best fit to data, as revealed by significantly smaller AIC scores as compared to the canonical model [*t*(33) = 2.457, p = 0.019]. The fit of the approach/avoidance model to behavioural data is shown in Fig 3C. A mixed-effects ANOVA with factors Group (approach, avoidance learner) and Learning rate (*α*_*Approach*_, *α*_*Avoid*_) revealed a significant Group x Learning rate interaction [F(1,32) = 9.049, p = 0.005], but no significant main effects [both p-values > 0.320]. Approach learners displayed relatively larger *α*_*Avoid*_ as compared to *α*_*Approach*_ [*t*(20) = 2.404, p = 0.026] while avoidance learners displayed relatively smaller *α*_*Avoid*_ as compared to *α*_*Approach*_ [*t*(12) = 2.179, p = 0.050]. Moreover, as compared to avoidance learners, approach learners displayed marginally larger *α*_*Avoid*_ [*t*(33) = 1.880, p = 0.069] but *α*_*Approach*_ did not differ between groups [*t*(33) = 968, p = 0.340]. Finally, there was no significant difference in the exploration/exploitation parameter *β* [*t*(33) = 1.610, p = 0.117]. These results indicate that the expression of a particular approach/avoidance learning style depends on how quickly a particular type of information is learned (i.e. positive or negative). Seemingly paradoxical, approach learners updated information more quickly following negative, as compared to positive outcomes, while avoidance learners showed the reverse trend. However, as will be detailed further below, a slow learning rate is beneficial in stochastic settings where information needs to be slowly integrated across many trials.

#### Individual correlations

For exploratory purposes, individual correlations between the different measures of approach/avoidance learning, SPSRQ scores, and model-fitted learning rates are displayed in Table 4. Additionally, based on the suggestion of one reviewer, we also test the relationship between the two learning systems by calculating correlations between the learning rates of the WM and the Habitual learning systems (see Table 4). Note that uncorrected thresholds are reported, and that no correlations between category measures (i.e. approach/avoidance learning vs. SPSRQ scores, approach/avoidance learning vs. learning rates, or SPSRQ scores vs. learning rates) survive a Bonferroni-corrected significance threshold of 0.000476 (e.g. α = 0.05/number of correlations (105)). For this reason, these results are not discussed further and should be interpreted with caution.

**Table 4:** Correlations between approach/avoidance learning, SPSRQ scores, and learning rates. Mean ± SEM.

Parameter	1.	2.	3.	4.	5.	6.	7.	8.	9.	10.	11.	12.	13.	14.
**Approach/avoidance learning**														
**1. Select A**														
**2. Reject B**	0.04													
**3. Select A–Reject B**	0.67^***^	-0.62^***^												
**SPSRQ**														
**4. SR**	-0.09	-0.24	0.05											
**5. SP**	-0.38^*^	0.030	-0.33^•^	-0.20										
**6. SR-SP**	0.24	-0.14	0.29^•^	0.72^***^	-0.74^***^									
**Working memory (WM)**														
**7. *α*_*Approach*_**	0.02	-0.11	0.09	-0.01	0.01	0.06								
**8. *α*_*Avoid*_**	0.32^•^	0.02	0.25	0.15	-0.38^*^	0.31^•^	0.38^*^							
**9. *α*_*Approach*_ – *α*_*Avoid*_**	0.01	-0.14	0.03	0.05	0.17	-0.01	0.62^***^	-0.29						
**Habitual**														
**10. *α*_*Approach*_**	-0.06	0.22	-0.20	-0.13	0.14	-0.16	-0.27	-0.01	-0.19					
**11. *α*_*Avoid*_**	0.31^•^	-0.07	0.32^•^	-0.39^*^	-0.21	-0.13	0.07	0.33^•^	-0.22	0.26				
**12. *α*_*Approach*_ – *α*_*Avoid*_**	-0.24	0.26	-0.40^*^	-0.01	0.44^**^	-0.30^•^	-0.22	-0.16	-0.06	0.61^***^	-0.39^*^			
**WM–Habitual**														
**13. *α*_*Approach*_**	0.04	-0.26	0.22	0.18	-0.20	0.28	0.70^***^	0.21	0.45^**^	-0.75^***^	-0.16	-0.59^***^		
**14. *α*_*Avoid*_**	0.02	0.15	-0.13	0.34^•^	-0.02	0.24	0.30^•^	0.36^*^	0.08	-0.29^•^	-0.62^***^	0.31^•^	0.26	
**15. *α*_*Approach*_ – *α*_*Avoid*_**	0.23	-0.23	0.39^*^	-0.15	-0.11	0.04	0.43^*^	-0.02	0.44^*^	-0.47^***^	0.32^•^	-0.80^***^	0.66^***^	-0.39^*^

“Working memory” and “Habitual” refers to the two different learning systems which can be assessed by fitting model parameters to behavioural data during the training and testing phase, respectively [12].

• p < 0.10

* p < 0.05

** p < 0.01

*** p < 0.001

## Discussion

The present study used a probabilistic selection task (PST; [1]) in combination with trait questionnaires to study the relationship between individual approach and avoidance learning styles and motivational traits pertaining to the general expression of approach and avoidance behaviours. Additionally, a computational approach was adopted in an attempt to elucidate the computational mechanisms mediating individual differences in learning styles. The results are discussed in detail below.

### Learning to approach and to avoid relate to individual expression of approach and avoidance motivational traits

Approach learners, i.e. participants that were relatively better at selecting the most rewarded A symbol as compared to rejecting the most punished B symbol in the testing phase, displayed increased trait approach motivation (SR), but decreased trait avoidance motivation (SP), as compared to avoidance learners.

These results show that biases between approach and avoidance learning relate to individual approach and avoidance traits, thus confirming the elusive link between individual motivational traits and learning styles, as illustrated by previous inconsistent findings. For example, using a scale designed to measure an individual’s risk for drug addiction, an unpredicted positive correlation between avoidance learning in the PST and novelty seeking, a trait commonly associated with approach behaviours, was recently reported [15]. By contrast, another study reported a positive correlation between avoidance learning and harm avoidance [4], and yet another failed to find significant correlations between approach and avoidance learning and traits using the BIS/BAS scales and a PST [16]. Here, we found that the SPSRQ was a better predictor of biases in approach-avoidance learning as compared to the BIS/BAS scales, even though both scales were designed to estimate the activation of the same two separate systems. However, while these scales are correlated, there are also indications of differences between them. In particular, as compared to the original BIS/BAS scales, the SPSRQ is a more recent attempt to specifically isolate the contribution of the impulsivity and the anxiety dimensions believed to drive the BAS and the BIS, respectively [24], and it has been suggested that the SPSRQ provides a better estimate of the BIS/BAS systems [30]. Moreover, similar to the present study, it was recently reported that the SPSRQ, but not the original BIS/BAS scales, correlated significantly with behavioural measures of approach motivation [31]. Additionally, in a recent study, no correlations between approach and avoidance learning in a PST and the BIS/BAS scales were reported [16]. Our data therefore add support to the suggestion that the SPSRQ may better capture key trait dimensions that relate to distinct behavioural dispositions, including approach and avoidance learning styles.

By demonstrating that the balance between approach and avoidance trait motivation relates to the balance between approach and avoidance learning, but not overall performance, our results suggest that improved learning of trait-congruent information may impede learning of other (trait-incongruent) information. The maintenance of motivational behavioural predispositions by such a mechanism would also account for the separate previous observations that participants displaying high trait optimism show strong deficits in learning information that is worse than expected, i.e. information inconsistent with their optimistic predisposition [32], that high trait anxiety increases fear acquisition but impedes fear extinction [33], and that high sensitivity to social rejection prevents extinction of conditioned responses to angry faces [34]. Thus, trait-like dispositions may influence learning so as to reinforce and maintain trait-congruent information which could lead to the continued expression of behavioural biases.

In relation to this notion, recent reports indicate that individuals displaying particular traits have an increased risk of developing mental and behavioural disorders [33,35], and that such disorders may develop and be maintained through biased learning processes [33,36]. Clarifying the factors that contribute to biases in approach and avoidance learning, and their relationship to behavioural predispositions and traits, may therefore aid us in understanding why some individuals are at a greater risk of developing disorders, in particular those associated with extreme expressions of approach and avoidance behaviours such as depression, anxiety, and addiction. The PST may be particularly well suited for this purpose because it has previously been used to highlight factors contributing to individual differences in reinforcement learning and decision making, including genetics [2,12], aging [9], pharmacology [1,3], dopamine receptor availability [10], and neuropsychiatric conditions [1,37].

Finally, only positive and negative (but no neutral) feedback was provided in the present study. This limitation, i.e. the absence of a neutral condition, makes it difficult to determine how approach and avoidance learning relates to the learning of other types of information. For example, it is unclear whether a particular learning style (i.e. approach learning) is associated with reduced learning of specifically trait-incongruent information (i.e. negative information), or all types of trait-irrelevant information (i.e. negative and neutral information).

### Approach and avoidance learning styles relate to differences in learning rates following positive and negative outcomes

Recent computational approaches suggest that two systems contribute to approach and avoidance learning [12,14]. The first system is related to the rapid updating of reward information in WM, while the second system is related to the slow integration of reward information and habitual responding [14]. It has been suggested that fitting computational models to behavioural data during training and testing phases of the PST, respectively, provides a means to gain insights into the functioning of the WM and habitual systems [12].

#### Approach and avoidance learning in working memory

Approach and avoidance learners did not display any differences in computational parameters when models were fit to behaviour during the training phase, i.e. to assess approach and avoidance learning in the WM system [12]. This finding is in-line with previous results showing that participants with different polymorphisms of the DARPP-32 and the DRD2 genes displayed different approach and avoidance learning styles, but did not differ in model-derived learning rates associated with the WM system [12]. The role of the WM learning system may be related to adapting behaviour on a relatively short time-scale by maintaining recent reward information in the WM. Indeed, polymorphisms of the COMT gene were associated with both differences in WM dependent learning rates following negative feedback, and the ability to switch responses following negative outcomes, but not with differences in approach and avoidance learning [12].

Another explanation may be related to the fact that A and B symbols were always presented as pairs within the same trials during the training phase. Thus, it is not clear whether increased selections of the A symbol are due to approach learning (i.e. increased selections of the frequently rewarded A symbol) or avoidance learning (i.e. increased rejections of the frequently punished B symbol). A model that was fit solely to the training data may therefore not be able to capture individual differences in approach and avoidance learning. However, this could be accomplished through paradigms which use separate approach and avoidance trials during the training phase [4,38,39].

#### Habitual approach and avoidance learning

It has been suggested that the ability to discriminate between subtle reward probabilities accumulated across many trials is more likely to involve the striatum of the basal ganglia, which integrates long-term probabilities of positive and negative outcomes through incremental changes in synaptic plasticity [13,14]. Indeed, when the models were fit to the testing phase, i.e. to assess approach and avoidance learning in the system related to habitual responding [12], approach learners showed relatively smaller learning rates following positive (vs. negative outcomes), while avoidance learners showed the reverse trend.

These findings, indicating that approach and avoidance learners respectively update information associated with positive and negative feedback more slowly, seems counter-intuitive to the results that approach and avoidance learners display better performance on symbols associated with frequent positive (i.e. select A) and negative outcomes (i.e. reject B), respectively. However, while large learning rates, which put emphasis on the most recent outcomes, are beneficial in deterministic contexts where outcomes closely correspond to a symbol’s true value, they could impede performance in more stochastic settings, i.e. during probabilistic feedback, because information needs to be integrated across many trials. For example, a learning rate of 1 takes into account only the most recent outcome and is optimal when a symbol is yoked to one specific outcome, i.e. when selecting a symbol yields 0 or 100% positive outcomes. By contrast, ignoring all previous reward history in a setting with probabilistic feedback causes large fluctuations in the representation of a symbol’s true value and therefore also suboptimal decision making [12]. Small learning rates are therefore beneficial for discrimination performance in the present study using probabilistic feedback. Specifically, better integration of positive outcomes across trials, as indicated by a small *α*_*Approach*_, enhances discrimination performance for stimuli associated with frequent positive outcomes, such as the most frequently rewarded A symbol. Conversely, emphasizing only the most recent history of negative outcomes, as indicated by a large *α*_*Avoid*_, impedes discrimination for stimuli associated with frequent negative outcomes, including the most frequently punished B symbol.

Whether model-derived learning rates display trait-like characteristic or vary across different settings is still unclear. For example, low learning rates for negative information could result in more accurate and stable representations of aversive memories and avoidance behaviours, thus contributing to the increased expression of avoidance-related predispositions and traits. However, small learning rates could also cause performance deficits when the encoding duration is limited or when stimulus-outcome contingencies are changing. While trait-like characteristics of individual learning rates have not received a lot of attention, some evidence suggests that people adapt their learning rates based on the volatility of the context, such that learning rates are large and small in contexts with high and low volatility, respectively [40]. Interestingly, the ability to regulate learning rates in an aversive context was related to the individual expression of trait anxiety [41]. Specifically, high trait anxiety was associated with a reduced ability to regulate the learning rates as a function of contextual volatility, as evidenced by a smaller difference between learning rates in volatile as compared to stable contexts. In the present study, negative learners displayed improved avoidance learning in a context with probabilistic feedback due to small *α*_*Avoid*_, and increased SP, a trait significantly correlated with trait anxiety [24]. It could therefore be predicted that trait anxiety should be specifically associated with performance deficits in a volatile context where fluctuations in reward contingency occur rapidly and large learning rates are beneficial. However, no significant correlation was detected between trait anxiety and the separate learning rates in volatile and stable contexts [41], therefore suggesting that trait anxiety relates more specifically to an inability to *adapt* learning rates in volatile aversive contexts. Yet, the present study suggests that the expression of different learning styles also depends on the differential learning rates associated with different outcomes (i.e. positive and negative feedback), an aspect which was not investigated in the previous study [41]. Thus, it remains an open question as to which extent learning rates display trait-like characteristics.

## Conclusion

The present findings demonstrate that inter-individual differences in approach/avoidance learning styles are tightly linked to motivational traits pertaining to approach/avoidance behaviours. Of note, due to the correlational nature of our data it cannot be concluded whether traits may modulate learning, or whether specific learning styles determine the expression of behavioural predispositions. Yet, these results are suggestive of a self-reinforcing process acting to increase the expression of behavioural biases, which could contribute to the gradual development of extreme beliefs and behaviours associated with mental and behavioural disorders. Moreover, the present findings imply that standard measures of trait motivation are indicative of individual learning strategies, and may thus serve to guide individually tailored educational programs, whose implementation could benefit from the recent advances in educational technology and e-learning.
