# Correction: Linking Individual Learning Styles to Approach-Avoidance Motivational Traits and Computational Aspects of Reinforcement Learning

**DOI:** 10.1371/journal.pone.0172379

**Published:** 2017-02-13

**Authors:** Kristoffer Carl Aberg, Kimberly C. Doell, Sophie Schwartz

The first author’s name is incorrect in the citation. The correct citation is: Aberg KC, Doell KC, Schwartz S (2016) Linking Individual Learning Styles to Approach-Avoidance Motivational Traits and Computational Aspects of Reinforcement Learning. PLoS ONE 11(11): e0166675. doi:10.1371/journal.pone.0166675
